# Mutant neutrophils and NETosis in clonal hematopoiesis: an emerging contributor of thromboinflammation and organ dysfunction

**DOI:** 10.1007/s11239-026-03290-8

**Published:** 2026-04-29

**Authors:** Muhammad Shaheer Mannan, Muhammad Waqas Khan, Muhammad Abdul Haseeb Khan, Ahmed Javed, Syed Mujtaba Hussain, Waleed Ahmad

**Affiliations:** 1 Department of Internal Medicine, Marshfield Clinic, Marshfield, Wisconsin USA; 2 Department of Internal Medicine, Medical University of South Carolina, Charleston, South Carolina USA; 3 Department of Internal Medicine, Ayub Medical College, Abbottabad, Pakistan; 4 Department of Internal Medicine, Lahore Medical and Dental College, Lahore, Pakistan

**Keywords:** Clonal hematopoiesis, NETosis, Thromboinflammation

## Abstract

Age-related proliferation of indeterminate capacity hematopoietic stem and progenitor cells bearing somatic mutations, mainly in DNMT3A, TET2, and ASXL1, is referred to as clonal hematopoiesis of indeterminate potential (CHIP). In addition to the well-known premalignant effects, CHIP causes systemic effects and predisposes to cardiovascular disease, thromboembolism, and dysfunction of multiple organs. Mutant neutrophils have been increasingly recognized as contributors to this pathology. These cells exhibit aberrant phenotypes and epigenetic alterations associated with pro-inflammatory and pro-thrombotic phenotypes that may favor NET formation. Uncontrolled NETosis may promote endothelial damage, platelet aggregation, and microvascular thrombosis that creates a vicious loop of thromboinflammation. The association of CHIP-related NETosis with venous and arterial thromboses, ischemic stroke, myocardial infarction, and organ-specific damage has been reported, although the main contribution is largely referred from indirect biomarker-based evidence or extrapolation from related conditions. Instead of detecting NET production directly, several studies employ indirect NET markers (such as MPO-DNA, citH3, and cfDNA). Activation of PAD4, generation of reactive oxygen species and inflammatory cytokines are the main modulators implicated in mechanistic studies. Clinically, therapies against NETs using DNase, PAD4, or cytokine blockade have the potential to reduce the risk of thromboinflammation. Although supported by compelling preclinical and observational evidence, there are still challenges because of the use of retrospective studies, murine models, and indirect measure of NET.The current evidence base consists primary of preclinical (murine and in vitro) studies along with observational human data. Future studies are required on prospective trials, mutation-directed biomarkers, and precision medicine modalities to regulate mutant neutrophil functions. Insight into NET-mediated pathogenesis in CHIP does not just clarify the pathways between clonal hematopoiesis and thromboinflammation and dysfunction in the body but also provides opportunities to formulate specific solutions to decrease cardiovascular and systemic illnesses in victims.

**Graphical Abstract:**

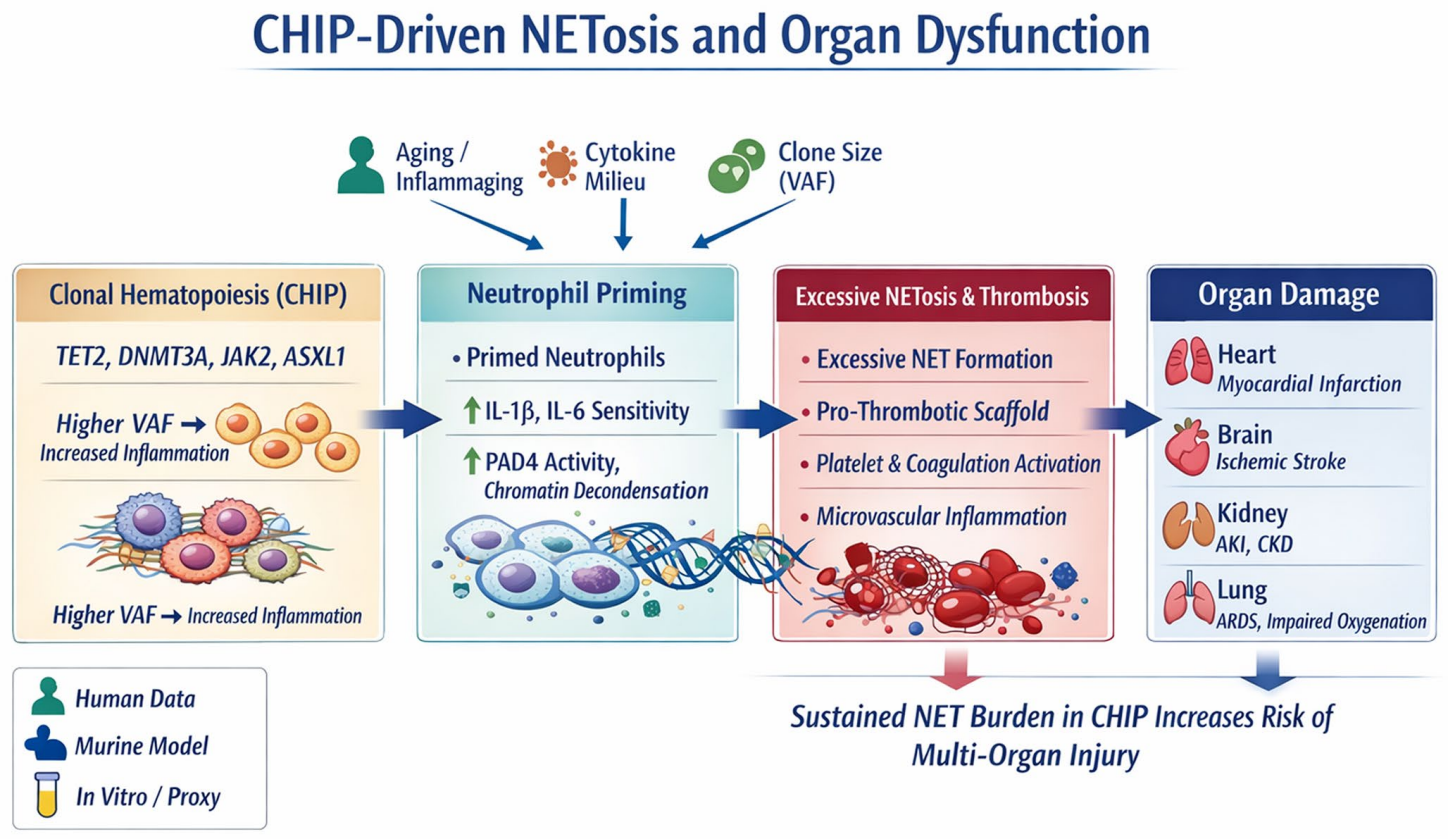

## Introduction

CHIP is an age-related clonal disorder that is characterized by the expansion of hematopoietic stem and progenitor cells with observable somatic mutations, but without apparent hematological malignancy. These cells constitute a genetically differentiated subset, deriving as a single founding cell, and defined by some distinct mutation in genes such as DMNT3A, TET2, or ASXL1 [1]. The overall frequency of CHIP, a condition characterized by somatic mutation linked to myeloid malignancies (primarily DNMT3A, TET2 and ASXL1) in the absence of a myeloid neoplasm, rises with age, reaching more than 10 per cent at age above 70 years old [2]. These mutations enable mutant cells to outcompete normal cells leading to amplified production of dysfunctional myeloid cells [3]. The current evidence base regarding NETosis in CHIP consists primarily of preclinical (murine and in vitro) studies along with observational human data with limited direct validation.

Clonal hematopoiesis comprises several related but separate entities. ARCH (age-related clonal hematopoiesis) broadly describes somatic mutations associated with age. These mutations frequently do not have any clinical ramifications. Similar mutations without cytopenias or cancer and with a measurable risk of progression are referred to as CHIP. While mutations with persistent cytopenias and higher progression risk are referred to as CCUS (Clonal Cytopenia of Undetermined Significance). Myeloproliferative neoplasms (MPNs) are full-blown blood malignancies. Although MPNs vary from CHIP physiologically, they are used in some studies to model CHIP.

Although CHIP was initially recognized for its role in malignancy, extensive epidemiological and mechanistic evidence now implicates that CHIP may contribute to chronic systemic inflammation and multiple age-associated diseases, particularly cardiovascular disease, thromboembolism, and organ dysfunction [4]. Neutrophils, the most abundant circulating innate immune cells, are emerging as potential contributors in thromboinflammatory processes. When neutrophils are activated, they undergo neutrophil extracellular trap (NET) formation, a specialized form of effector response in which decondensed chromatin and granular proteins are extruded into the extracellular environment, forming web-like structures that immobilize pathogens. While NETs serve antimicrobial roles, excessive or dysregulated NETosis promotes thrombus formation, endothelial injury, and enhances inflammatory responses in multiple pathologies.

Despite this, NETosis has been addressed only peripherally in the CHIP literature, with the focus on monocyte/macrophage inflammasome activation and systemic cytokine elevation instead of neutrophil dysfunction and NET-mediated immunothrombosis. NETosis is treated as a side effect of inflammation, not solely as a mutation-driven process caused directly by altered neutrophils. This narrative review focuses on mutant neutrophils and dysregulated NETosis as a potential contributing mechanism linking clonal hematopoiesis to thromboinflammation and organ dysfunction.

## Clonal hematopoiesis and myeloid bias

### Definition and genetics of CHIP

Clonal hematopoiesis of indeterminate potential (CHIP) is defined by the clonal expansion of hematopoietic stem cells carrying leukemogenic mutations in individuals without hematologic malignancy, dysplasia, or cytopenia. Roughly 80% of individuals with CHIP involve mutations in epigenetic regulators DNMT3A, TET2, ASXL1, DNA damage repair genes PPM1D, TP53, the regulatory tyrosine kinase JAK2, or mRNA spliceosome components SF3B1 and SRSF2 [4].

DNMT3A encodes a DNA methyltransferase, and it may enhance the self-renewal of hematopoietic stem and progenitor cells by impairing de novo methylation [5]. TET2 regulates DNA demethylation through oxidation of methylcytosine, and TET2 mutations impair normal differentiation, particularly under inflammatory conditions as shown in experimental models [6]. Mutations in ASXL1 interfere with chromatin modification and are associated with more aggressive clonal behavior and elevated progression risk which have been observed in clinical cohorts and preclinical studies.

Variant Allele Frequency (VAF) is the key parameter used to define CHIP, representing the proportion of sequencing reads that carry a mutation. CHIP is typically defined at a VAF of 2% or higher, although lower-frequency clones may still be biologically relevant [7]. Importantly, VAF indicates the relative proportion of the mutant clone within the hematopoietic compartment but does not correspond to the severity of the disease. It is an indication of the prevalence of a mutation at a given genomic site. Its predictive power of prognosis and treatment response has been studied, but this technology cannot be used in clinical practice due to a lack of standard thresholds and inconsistency of sequencing results [8].

A clear distinction between CHIP and myelodysplastic syndrome (MDS) is important both in medical and biological terms. Although CHIP and MDS have some common genetic mutations, the two diseases differ; MSD is characterised by chronic cytopenias, bone marrow dysplasia, and inefficient production of blood cells, as well as a significant risk of having acute myeloid leukemia. Conversely, individuals with CHIP possess normal blood counts and no morphological anomalies. Therefore, CHIP may be a premalignant or risk condition, and not a hematologic malignancy, an intermediate between normal aging hematopoiesis and overt myeloid disease [9].

### Myeloid skewing in CHIP

The primary characteristic of CHIP is myeloid skewing where mutant hematopoietic stem and progenitor cells produce the majority of myeloid lineage cells. These clones have a growth and survival advantage, gradually expanding over time [10]. Such clonal proliferation largely involves the myeloid lineage, with neutrophils and monocytes more often derived from mutant clones more than lymphoid cells [11]. Alteration of the normal differentiation processes through mutations in genes associated with the disease, including TET2, DNMT3A, and ASXL1, may cause hematopoiesis to shift to the myeloid lineage [12]. As a result, the circulating neutrophils and monocytes tend to be of the mutant clone, and this depicts lineage bias.

Myeloid skewing in CHIP is not purely a quantitative process but also has functional modifications that influence innate immune responses. These cells can exhibit increased inflammatory activity, including increased production of pro-inflammatory cytokines and enhanced responsiveness to immune stimuli [13]. Therefore, CHIP-associated myeloid skewing can not only increase the number of myeloid cells but can also alter their function [14]. This combination links clonal hematopoiesis to immune imbalance and provides a basis for its association with age-related inflammatory disorders, highlighting that CHIP is a biologically active state rather than a benign age-related finding [15].

Key CHIP mutations, their effects on neutrophil function, NETosis, and clinical implications are summarized in Table 1.

**Table 1:** Key CHIP mutations, effects on neutrophil function and NETosis, and clinical implications

Mutation	Effect on neutrophil	NETs observed	Evidence type	Model/ Cohort	Reference
TET2	↑Inflammatory signaling, epigenetic priming	↑ NET formation	Direct NET imaging / MPO-DNA	Murine knockout & human ex vivo	[18, 19, 29]
DNMT3A	↑ Cytokine production, ROS	↑ NET formation	Surrogate markers (MPO-DNA, citH3)	Human ex vivo, murine	[20, 29]
JAK2V617F	↑Thrombogenic phenotype	↑ NET formation (basal & stimulated)	Surrogate markers	Murine transgenic, some human observational	[21, 36]
ASXL1	Epigenetic dysregulation	Limited / unknown	Proxy / inferred	Mostly murine	[12, 15]

## Neutrophil biology in CHIP

Clonal hematopoiesis of indeterminate potential (CHIP) develops due to the clonal expansion of hematopoietic stem cells with somatic mutations, predominantly involving the epigenetic regulators TET2 and DNMT3A. Although the monocytes are regarded as the main mediators of the CHIP-associated inflammation by many researchers, emerging data from murine models and in vitro studies suggests that neutrophils may be functionally reprogrammed and that they may contribute to vascular inflammation and thrombotic events. Consequently, these observations highlight that neutrophils are not entirely passive and may participate in CHIP-associated inflammation, though human data and confirmation is limited [16, 17]. 

### Altered neutrophilic phenotype

CHIP-mutant progenitors produce such neutrophils that exhibit mutant gene expression profiles that may favour the inflammatory pathway. The mutations in TET2 and DNMT3A cause epigenetic dysregulation, which may result in the upregulation of pathways related to NF-κB signaling, cytokine production, inflammasome activation, and NET formation and may occur without acute infection in preclinical models [18, 19]. This epigenetically “primed” state may lower the threshold for the inflammatory responses, which may potentially contribute to chronic low-grade inflammation, a prime characteristic of CHIP-associated CVS diseases. Moreover, some studies report increased activations of neutrophils in CHIP-positive individuals, which may reflect that these changes are intrinsic to neutrophil biology rather than secondary to systemic inflammation alone [20].

CHIP-associated neutrophils promote increased cytokine signaling. The levels of IL-1β and IL-6 may be elevated in the circulating blood of CHIP patients, which can promote endothelial activation, leukocyte recruitment, and platelet aggregation. PAD4 activity and intracellular calcium influx are greatly enhanced by the release of IL-1β signaling and excess cytokine which may enhance NET formation as suggested by preclinical and in vitro studies; human evidence is limited [21].

The functionally altered neutrophils may increase the production of Reactive Oxygen Species (ROS). Increased oxidative metabolism might weaken the nuclear membrane and will aid the nuclear translocation of neutrophil elastase (NE) and myeloperoxidase (MPO), and may facilitate in chromatin decondensation during the process of NETosis [22]. The Reactive oxygen species (ROS) may contribute to damaging the endothelium, oxidizing the lipids, and promoting the coagulation pathways, suggesting a strong link between neutrophil dysregulation and thrombotic risk [23].

### Priming for NETosis

The clonal hematopoiesis of indeterminate potential has neutrophils with primed phenotypes that may exhibit a low activation threshold for the process of NET formation. Normally, the triggers for NETosis are strong microbial or inflammatory stimuli, but any weak physiological activity, such as platelet interaction, may be potent enough to stimulate the neutrophils derived from CHIP. Human data are limited, and evidence for continuous low-level NETosis in vivo remains preliminary [24].

The neutrophils undergo apoptosis under normal conditions, and then they are removed by macrophages so that the inflammation response can end. In CHIP, neutrophils may form NETs [25]. Various components of NETs, such as histones and extracellular DNA, can persist in tissues and circulation, potentially emitting signals that may promote the activation of endothelial cells, macrophages, and platelets. This might extend the inflammatory process and stabilize the thrombus [26]. The neutrophil remains primed in CHIP due to epigenetic dysregulation. During hematopoiesis, the changes in chromatin structure may stabilize inflammatory and NET gene programs that remain active in neutrophils even though they have a short life span. These observations explain how these somatic mutations lead to persistent immune problems. These mechanisms support the notion that CHIP-related neutrophils don’t remain passive, but they may contribute to inflammation and thrombosis.

## NETosis: mechanisms and regulation

Neutrophil extracellular traps (NETs) are web-like structures that neutrophils produce, which are composed mostly of DNA and enzymes like myeloperoxidase (MPO), neutrophil elastase (NE), and cathepsin G. Even though NETs function is to assist in the capture and eradication of microbes, excessive and uncontrolled NET formation can contribute to platelet activation that leads to blood clot formation, injury to blood vessels, and major organs [27]. This is especially significant in chronic inflammatory disorders such as clonal hematopoiesis of indeterminate potential (CHIP) where early evidence from human studies and experimental models suggests potential contribution to vascular pathology.

### Overview of NETosis

Two major forms of NETosis have been described: suicidal NETosis and vital NETosis.

In suicidal NETosis, neutrophils undergo chromatin decondensation, nuclear envelope rupture, and plasma membrane collapse resulting in neutrophil death and the release of nuclear DNA components outside the cell. This process depends on NADPH oxidase–mediated ROS production and is stimulated by strong stimuli [27].

In contrast, vital NETosis enables neutrophils to release DNA while maintaining membrane integrity and limited functional capacity, thereby preventing immediate cell death. This type of NETosis occurs when neutrophils come in contact with platelets or when the complement system is activated. Contrary to suicidal NETosis, vital NETosis does not depend on NADPH oxidase–mediated ROS production [28].

A fundamental molecular step in NET formation is chromatin decondensation which is chiefly regulated by protein arginine deiminase 4 (PAD4). PAD4 catalyzes the histone arginine residue conversion to citrulline, which weakens histone and DNA binding and results in chromatin losing its tight structure. This process is facilitated by the ROS production that leads to membrane permeabilization that then allows entry of enzymes like NE and MPO into the nucleus further weakening chromatin structure. Much of this mechanistic insight comes from experimental models, with limited direct human neutrophil data.

Other pathways that regulate NETosis involve mitochondrial ROS formation, calcium signaling and gasdermin D-mediated porosification [28]. Regulation of NETosis by these varying pathways highlights the reliance of NETosis to varying stimuli.

### Enhanced NETosis in mutant neutrophils

CHIP experimental work, mostly in murine models, suggests that mutations in genes like TET2 and DNMT3A have a direct potentiating effect on the formation of NET. Ex vivo studies also suggest that these mutated neutrophils produce excessive NETs in the baseline conditions and in the response to various stimuli [29]. These human studies largely report surrogate NET markers (MPO-DNA, citH3, cfDNA) rather than direct NET visualization.

Inflammatory signaling has an additional effect of increasing NET generation in CHIP. Evidence from murine studies suggest that upon activation of the NLRP3 inflammasome, IL-1β maturation is caspase-1 dependent, and it prepares neutrophils to produce ROS and activate PAD4. The NETs themselves have the capacity to induce the release of other inflammatory cytokines, including IL-1β and IL-6, by immune and endothelial cells. These cytokines increase platelet activity shifting the hemostatic balance towards coagulation which creates a self-reinforcing loop of inflammatory mechanism.

NETs are structural scaffolds in which platelet aggregate, fibrin deposition and red blood cells become trapped. This leads to thickening of thrombi since activation of intrinsic pathways occurs by NET-associated histones. Pharmacologic inhibition of PAD4 or enzyme degradation of NETs with DNase in experimental model environments significantly decreases the thrombus load, indicating that NETosis may be a future therapy (CHIP-related vascular disease) [29].

## NETosis as a driver of thromboinflammation

Thromboinflammation refers to the pathological process of two-way reciprocal activation and amplification of inflammation and blood clotting (thrombosis). The inflammation leads to the activation of the immune system that, in turn, facilitates coagulation and vascular damage. The inflammation results in the formation of clots, which in turn contributes to further inflammation resulting in a vicious cycle [30]. Neutrophil extracellular traps (NETs) have come out as initiators of this process, with evidence from murine models and limited human studies. Neutrophil stimulation leads to the release of inflammatory signals and NETs. In murine models, these NETs promote platelets and clotting factors resulting in the development of a thrombus, while ex vivo studies in CHIP-positive human neutrophils suggest a similar but less potent prothrombotic effect [31].

Although the NETs play a critical role in trapping and neutralizing the pathogens and microbes, abnormal or excessive NETosis may lead to significant pathological consequences. There is evidence to show that NETs proactively trigger, promote, and stabilize the formation of thrombus in the venous and arterial systems [32]. In the context of clonal hematopoiesis, murine studies suggest that neutrophils with mutations such as TET2 or DNMT3A may exhibit exaggerated NET formation, and limited ex vivo human data support a potential prothrombotic and proinflammatory state.

### NETs and coagulation

One of the proposed mechanisms in which NETs facilitate thrombosis is that they act as a physical scaffold in the development of thrombus. Experimental studies suggest that NETs assembly can occur without explicit endothelial injury because of the structural framework of NETs [33]. This suggests that NETs may trigger and maintain the formation of thrombus at the location of injury, and not only inflammation. In addition to acting as structural cascades, NETs may also act as coagulation cascades, where tissue factor (tf), the major initiator of the extrinsic coagulation pathway, plays an important role.

#### Tissue factor expression

In experimental models, it has been shown that Tf expressed by neutrophils in the presence of Tf-bearing microparticles or by direct neutrophil expression in NETosis is incorporated in the NET structures. When exposed, Tf binds factor VIIa, which activates factor X, releases thrombin, and causes fibrin formation [34]. NET-associated Tf may link the innate immune activation to coagulation.

Moreover, experimental models suggest that histones and neutrophil proteases, which are constituents of NETs, have the capacity to trigger activation of certain endothelial cells and monocytes, which may further promote coagulation [35]. This may form a self-reinforcing cycle where inflammation leads to the formation of NETs, NETs contribute to coagulation, and further processes of clotting fuels further inflammation. In clonal hematopoiesis, the cycle can be reinforced by promoting a more sustained NET release, although human evidence remains limited.

#### Platelet–neutrophil interactions

Platelet neutrophil interactions may contribute to NET-mediated thromboinflammation. Neutrophils interact with activated platelets via adhesion molecules like P-selectin and PSGL-1, leading to intracellular signaling pathways and also through soluble mediators including platelet factor 4 and high-mobility group box 1 (HMGB1), which amplify neutrophil activation and formation of neutrophil extracellular traps (NETs) [36]. Studies from murine models suggest that NETs, once exposed, can reciprocally activate platelets by exposing histones and DNA that function as potent platelet agonists, thereby promoting platelet aggregation and thrombus stabilization [37].

### Clinical thrombotic associations

#### Venous thromboembolism

The establishment of venous blood clots, including pulmonary embolism and deep vein thrombosis has been associated with NETosis. Experimental and some human studies show that these clots commonly contain NETs, which entrap fibrin and blood cells, providing them with a mesh or a framework that keeps the clot together [38]. The degradation of NETs by enzymatic or pharmacological activity has been shown in animal models to reduce the size of thrombus and dissolve the clot.

Higher levels of circulating surrogate NET markers (e.g., DNA fragments and NET-related proteins) released during NET formation are linked to a higher risk of venous clot formation and disease occurrence. Chronic low-grade inflammation, being a salient feature of clonal hematopoiesis, may promote increased NETs release by neutrophils [39]. While large human studies directly connecting CHIP to VTE are still limited, the way NETs promote clot formation provides a plausible biological explanation for how this risk could occur.

#### Arterial thrombosis

In arterial thrombosis, NETosis may contribute to the destabilization of atherosclerotic plaques as well as blood clot formation. Studies in human plaques and experimental models have identified NETs whose components, such as histones and neutrophil enzymes, may cause smooth muscle death and break down the structural framework of the plaque, which makes it unstable and more likely to rupture [40]. The DNA and proteins in NETs provide a surface that promotes thrombin production and fibrin deposition, creating dense clots that are harder to break down. Clonal hematopoiesis may further influence this process. Mutations in blood cells make neutrophils more likely to release NETs and cause inflammation. However, direct human evidence linking CHIP-associated NETosis to thrombotic outcomes remains limited, and much of the mechanistic understanding is derived from experimental models.

#### Stroke and myocardial infarction

NETosis has been implicated in ischemic stroke and myocardial infarction. Human studies have identified that NETs have been found in clots of patients of acute ischemic stroke and that higher NET levels may cause worse neurological outcomes. NETs may block small blood vessels after clot-dissolving treatment that prevent proper blood flow from being restored and thereby contributing to ischemia and secondary tissue injury as well.

Similarly, in myocardial infarction, NETs have been detected at blocked coronary arteries and may contribute to vascular damage by injuring blood vessels and blocking small vessels in the heart. Higher levels of NETs in blood are associated with increased risk of cardiovascular disorders [41]. Given the already known association of clonal hematopoiesis to increase cardiovascular risk, NETosis provides a plausible biological explanation for how abnormal blood cells can contribute to clot-related damage in the heart and brain.

#### NET-mediated organ dysfunction

Besides the role of neutrophil extracellular traps (NETs) in thrombosis and inflammation, it may also contribute to organ dysfunction by mechanisms like that of microvascular obstruction, prolonged inflammation and endothelial damage. Blood flow at the microcirculatory level can be impaired due to excessive NET formation which can lead to dysfunction across multiple organ systems. Apart from the mechanical effects, NET components such as histones and neutrophil proteases may exert cytotoxic effects leading to endothelial injury and thereby causing increased vascular permeability. This may disrupt the barrier function of endothelial cells and therefore causes increased inflammatory effects [17]. In clonal hematopoiesis setting, mutations in hematopoietic stem cells keep the immune system overactive because of which the neutrophils may exhibit a primed response at all times. As a result they release excessive NETs which may lead to damage to blood vessels and tissue and thereby leading to organ dysfunction.

#### Cardiovascular system

In the cardiovascular system, NETs may contribute to the worsening of atherosclerosis and microvascular injury. Fatty plaques found in arteries have shown NETs, which may be responsible for increasing inflammation and making plaque unstable. NET components, such as histones and neutrophil proteases, may damage the inner lining of blood vessels during NETosis, which can reduce the production of nitric oxide and increase vascular permeability. These effects may accelerate plaque growth and make it more susceptible to rupture [17].

The other way in which NET leads to microvascular injury is through platelets and fibrin deposition on small vessels. This may lead to the formation of numerous tiny clots on a micro vascular level and these are able to decrease blood flow to the tissue without any huge clots. CHIP has also been associated with adverse cardiovascular outcomes as in individuals with clonal hematopoiesis, there is more endothelial dysfunction and microvascular thrombosis potentially related to increased NET burden.

#### Renal system

The kidney is particularly susceptible to the NET-mediated damage due to the high density of blood vessels in it. NETs are potentially harmful to the lining of these veins, and they may contribute to local inflammation and the formation of small clots in the kidney. Endothelial cells of the glomerulus and tubules may be susceptible to direct damage by NET elements, including histones and neutrophil proteins which raise the endothelial barrier permeability and subsequently facilitate intrinsic penetration of inflammatory cells into the kidneys [19, 42].

In addition, NETs may accord with the development of glomerular microthrombi, which lower renal perfusion and hinder filtration. This sustained impaired perfusion, coupled with fibrosis, may lead to chronic kidney disease. In clonal hematopoiesis, ongoing low-grade inflammation and increased NET release may make patients more prone to repeated kidney injury. This puts patients at higher risk of repeated and more frequent chronic kidney diseases, suggesting a potential link between CHIP and renal dysfunction.

#### Pulmonary and other organs

Excessive NET formation in the lung has been associated with increased vulnerability to acute respiratory distress syndrome (ARDS) and lung injury [20]. Impaired gas exchange, pulmonary edema, and worsening respiratory failure may result from damage to the epithelial and endothelial barriers of the lung due to the accumulation of NETs in alveolar gaps and pulmonary microvessels. The microthrombi associated with NET may further impair oxygenation and pulmonary blood flow [21].

NET-mediated endothelial injury may affect multiple organ systems in addition to the lungs. Widespread endothelial damage may promote microvascular dysfunction, contributing to multiorgan failure in severe inflammatory states. NETs in the circulation can exacerbate organ damage by acting as prothrombotic and proinflammatory stimuli at remote locations. In clonal hematopoiesis, sustained myeloid activation may contribute to increased systemic NET load, potentially raising the risk of organ failure and widespread endothelial damage. However, most of the evidence linking NETosis and organ dysfunctions is derived from observational and experimental studies, with limited data from CHIP-specific human populations.

Proposed thrombotic and organ-specific consequences of NET formation are summarized in Table 2.

**Table 2:** NET-mediated thrombotic and organ effects

System / Organ	NET Effects	Mechanisms	Clinical Consequences	Evidence Level
Venous system	NET scaffold formation in clots	DNA traps platelets & RBCs, histones activate coagulation	Deep vein thrombosis, pulmonary embolism	Human in vivo / Preclinical
Arterial System	NETs in plaques and thrombi	Endothelial damage, smooth muscle apoptosis, thrombin generation	Myocardial infarction, stroke, arterial thrombosis	Human in vivo / Preclinical
Heart/ Cardiovascular	Microvascular obstruction, endothelial injury	NETs trigger platelet aggregation & cytokine release	Heart failure progression, ischemic injury	Preclinical / Human in vitro
Kidney	Glomerular microthrombi, vascular injury	Endothelial damage, leukocyte recruitment, impaired perfusion	Acute kidney injury (AKI), CKD progression	Preclinical / Human in vitro
Lungs	Microvascular obstruction, inflammatory damage	NETs promote platelet aggregation, endothelial permeability	ARDS susceptibility, impaired gas exchange	Preclinical / Human in vitro
Systemic	Endothelial activation and inflammation	NETs circulate and stimulate immune cells, cytokine amplification	Multi-organ dysfunction, chronic thromboinflammatory state	Preclinical / Human in vitro

#### Clinical implications and therapeutic perspectives

CHIP is increasingly recognized as a contributor to thromboinflammatory risk and multisystem organ dysfunction. The discovery that myeloid mutant cells, especially neutrophils to be triggered, and NETosis may have potential clinical implications in their quest to conceptualize the risk stratification and treatment of an aging population.

### Risk stratification

#### VAF-driven monitoring and mutation specificity

Indeed, there is evidence from large genomic cohort studies that the cardiovascular risk conferred by CHIP is a dose-response phenomenon of the VAF and not a binary phenomenon. Clones that fulfil the VAF of 10% or more carry an increased hazard ratio of atherosclerotic cardiovascular disease, heart failure, and all-cause mortality than small clones [4]. This threshold may reflect a fundamental amount of mutated leukocytes needed to maintain systemic inflammation.

Furthermore, the genetic background is important; TET2, DNMT3A, and JAK2 mutations have been associated with higher inflammatory burden [37, 43]. Those results imply that a carrier of high-VAF clones may require closer monitoring of cardiovascular parameters and more aggressive treatment of conventional risk factors such as hypertension and hyperlipidemia despite the fact that the current clinical guidelines do not yet require universal screening.

#### Role of inflammatory biomarkers

As CHIP is associated with a chronic low-grade systemic inflammatory state, traditional risk calculators often greatly underestimate the danger to the patient. Mutant myeloid cells have been associated with overproduction of IL−1β and IL − 6, reflected in elevated levels of high-sensitivity C-reactive protein (hsCRP) [44]. In CHIP carriers, increased hsCRP may serve as a functional marker of inflammatory activity; higher levels have been associated with significantly worse prognosis following acute cardiac events [45]. Integration of these biomarkers with genomic data may eventually permit a “thromboinflammatory risk score” that identifies patients in whom early anti-inflammatory intervention would be beneficial.

### Targeting NETosis and immunothrombosis

#### DNase therapy

The identification of NETs as a pro-thrombotic scaffold has suggested new therapeutic possibilities. NETs can act as a physical scaffold that entraps platelets and activates the intrinsic coagulation pathway [46]. In experimental models it has been demonstrated that DNase I administration can degrade the DNA backbone with resultant destabilization of thrombi and reduced thrombus size in models of arterial and venous thrombosis. DNase is currently used for pulmonary clearance in cystic fibrosis; how it may function to dissolve thrombi in CHIP-associated injury remains an active area of investigation.

#### PAD4 inhibitors

Peptidylarginine deiminase 4 (PAD4) is a ker regulator of NET formation by catalyzing the citrullination of histones, which enables chromatin decondensation. In preclinical models it has been shown that pharmacologic inhibition or genetic deletion of PAD4 reduces the release of NETs and significantly reduces the incidence of immunothrombosis [47]. Targeting PAD4 presents a precision medicine strategy because it potentially disrupts the “sticky” NET scaffold potentially without inducing the global immunosuppression associated with broad-spectrum anti-inflammatory drugs.

#### IL-1 blockade and the NLRP3 pathway

The link between CHIP and the NLRP3 inflammasome represents one of the most clinically validated therapeutic targets. TET2 deficiency, in particular, has been associated with the hypersecretion of IL-1β. Results from the CANTOS trial evaluating the IL-1β inhibitor canakinumab demonstrated a significant reduction in recurrent cardiovascular events [44].

Post-hoc analyses demonstrated that the benefit was most pronounced in individuals with CHIP mutations, supporting a potential “proof-of-concept” that targeting the specific cytokine pathways activated by mutant clones can modify clinical outcomes [4, 44].

#### Antithrombotic considerations

Despite the compelling link between NETs and coagulation, the use of standard antiplatelet or anticoagulant therapies specifically based on CHIP status is not yet supported by RCT data. Since the architecture of a NET-rich thrombus is inconsistent with that of a typical fibrin-rich clot, the efficacy of the traditional agents or the susceptibility to bleeding in such patients can be low. This suggests that combination strategies may be explored in future studies. Importantly, no CHIP-specific clinical trials have yet validated these therapeutic approaches.

### Methodological considerations and limitations of current evidence

There are multiple methodological problems with CHIP-driven thromboinflammation, which makes it difficult to bring the fast-changing lab results into generalized clinical practices.

#### Predominance of observational and retrospective studies

The evidence base is predominantly composed of Observational and Retrospective Studies. The entirety of evidence relating CHIP to cardiovascular or organ-specific outcomes are all products of retrospective studies of large biobanks, including the UK Biobank and TOPMed. Although these research works offer strong statistical relationships, they are by nature descriptive in nature and may not fully account for all lifestyle, environmental, or socioeconomic confounding factors. This uncontrolled, prospective information constrains our capacity to designate definite causality to particular mutations as compared to the extensive inflammatory milieu in which they coexist [48].

#### Reliance on murine models and translational biology

Much of the mechanistic platform regarding the CHIP–NETosis axis is based on either Tet2-knockout or Jak2V617F transgenic mouse models. Inherent neutrophil physiology differences limit direct translation to human disease. Human peripheral blood is neutrophil-rich, comprising 50–70% of total leukocytes, whereas murine blood is lymphocyte-predominant, with neutrophils constituting only 10–30% [49]. Moreover, human neutrophils exhibit increased specific granules and dissimilar signaling kinetics regarding PAD4-mediated chromatin decondensation. These results suggest that “NETotic burden” in humans may be significantly more serious and sustained compared to how murine models illustrate this aspect currently.

#### Indirect Measurement of NETosis in Humans

Direct visualization of NET formation in human patients remains an elusive gold standard. Most clinical studies rely on circulating proxies, given the fragile and transient nature of these extracellular DNA structures. A limitation is that there is no standardized method to quantify intact NETs in clinical settings. Markers normally adopted have been Cit-H3 or MPO-DNA complexes that indirectly indicate NETosis; however, such biomarkers may be elevated in non-CHIP inflammatory states [50, 51].

#### Confounding by age and “Inflammaging"

CHIP is essentially a disease of the aging immune system. Teasing apart mutation-specific neutrophil hyper-reactivity from the baseline physiologic decline otherwise known as inflammaging represents a major challenge [50]. Chronic, low-grade systemic inflammation and age-associated comorbidities such as hypertension and diabetes commonly occur in the same demographic as CHIP carriers.

#### Heterogeneity in CHIP definitions and VAF thresholds

An additional challenge relates to the lack of a universal consensus on what constitutes a “clinically significant” clone. Studies range dramatically in terms of their Variant Allele Fraction (VAF) cutoffs, with ultra-deep sequencing at 0.1% and more traditional 2% or high-risk 10% cutoffs [52]. This generates a high degree of heterogeneity in the amount of hazard ratios reported and makes the task of researchers to perform accurate meta-analyses across dissimilar patient cohorts difficult.

### Future directions and research gaps

Addressing these gaps will require the shift of the field to precision medicine by filling a number of critical gaps prospectively by mechanistically diverse studies.

#### The transition to prospective clinical trials

There is a need for the prospective randomized trials. So far, we do not have any scientific evidence that screening for and treating CHIP-positive status itself prolongs survival in these patients. Further research should be focused on whether anti-inflammatory therapy with the use of IL-6 inhibitors or PAD4 antagonists reduces the incidence of MACE, particularly in high-risk mutation carriers [51, 53].

#### Development of precise “NET-Biomarkers

While current markers such as hsCRP have already demonstrated strong correlations with poorer outcomes in CHIP carriers post-cardiovascular events, the next stage is the development of more specific “CHIP-responsive” biomarkers. There is a need for diagnostic tools that can discriminate between normal neutrophil function and the “primed” NETosis state specifically induced by TET2 or JAK2 mutations. This would potentially enable a “liquid biopsy” approach to monitoring the inflammatory activity of a clone in real time.

#### Expanding to organ-specific outcomes

While cardiovascular disease remains the main focus, the contribution of mutant neutrophils to other organ systems is a growing area of interest. Recent data have started to implicate CHIP in acute kidney injury and more specific cardiac pathologies such as myocarditis and pericarditis [48].

Future studies should evaluate a role for NETosis in neuroinflammation (dementia), pulmonary fibrosis, and chronic kidney disease needs to be defined to determine if mutant neutrophils represent a systemic threat to all microvascular beds.

#### Precision medicine and genotype-directed therapy

The ultimate goal is a precision medicine algorithm where therapeutic choice is dictated by the exact mutation profile of the patient. TET2-mutant carriers may benefit from IL-1β or NLRP3 inhibitors due to their specific cytokine release profiles [49, 53]. JAK2-mutant carriers may be candidates for more aggressive antithrombotic management or specific kinase inhibitors to mitigate their unique prothrombotic neutrophil behavior. DNMT3A mutation carriers may need other immunomodulatory strategies targeting T-cell/neutrophil cross-talk. Importantly, integrating genotype, immune phenotype, and clinical outcomes into a unified model remains an ongoing challenge requiring prospective validation.

## Conclusion

Mutant neutrophils and increased NET production represent a potential contributing pathway connecting clonal hematopoiesis to thromboinflammation and organ failure. The abnormal myeloid cells resulting in CHIP may exhibit potential for excessive NETs formation, which may lead to clot formation in different components of the microvascular system and cause injury in different organs of the body. This causes a self-reinforcing loop whereby coagulation and inflammation compound each other. This may partly explain the increased risk of cardiovascular and multi-organ risk in the affected individuals. A conceptualization of NET-driven illnesses offers potential prospects of clinical therapy such as NET-directed therapy and risk stratification with biomarkers. Although existing evidence is largely observational and pre-clinical, additional clinical research in the future is necessary to determine specific therapies and interventions that could mitigate the pathological effects of thromboinflammation and enhance organ maintenance in patients with CHIP. Importantly, there is still no direct human causal evidence connecting CHIP-associated neutrophil dysfunction and NETosis to clinical outcomes.

## Data Availability

No datasets were generated or analysed during the current study.
